# Characterization and Localization of Citrullinated Proteoglycan Aggrecan in Human Articular Cartilage

**DOI:** 10.1371/journal.pone.0150784

**Published:** 2016-03-04

**Authors:** Tibor T. Glant, Timea Ocsko, Adrienn Markovics, Zoltan Szekanecz, Robert S. Katz, Tibor A. Rauch, Katalin Mikecz

**Affiliations:** 1 Section of Molecular Medicine, Departments of Orthopedic Surgery, Biochemistry and Internal Medicine, Rush University Medical Center, Chicago, Illinois, 60612, United States of America; 2 Department of Orthopedic Surgery, Rush University Medical Center, Chicago, Illinois, 60612, United States of America; 3 Department of Rheumatology, Institute of Medicine, University of Debrecen, Faculty of Medicine, Debrecen, H-4012, Hungary; 4 Rheumatology Associates, Rush University Medical Center, Chicago, Illinois, 60612, United States of America; Institute of Immunology, Rikshospitalet, NORWAY

## Abstract

**Background:**

Rheumatoid arthritis (RA) is an autoimmune disease of the synovial joints. The autoimmune character of RA is underscored by prominent production of autoantibodies such as those against IgG (rheumatoid factor), and a broad array of joint tissue-specific and other endogenous citrullinated proteins. Anti-citrullinated protein antibodies (ACPA) can be detected in the sera and synovial fluids of RA patients and ACPA seropositivity is one of the diagnostic criteria of RA. Studies have demonstrated that RA T cells respond to citrullinated peptides (epitopes) of proteoglycan (PG) aggrecan, which is one of the most abundant macromolecules of articular cartilage. However, it is not known if the PG molecule is citrullinated *in vivo* in human cartilage, and if so, whether citrulline-containing neoepitopes of PG (CitPG) can contribute to autoimmunity in RA.

**Methods:**

CitPG was detected in human cartilage extracts using ACPA+ RA sera in dot blot and Western blot. Citrullination status of *in vitro* citrullinated recombinant G1 domain of human PG (rhG1) was confirmed by antibody-based and chemical methods, and potential sites of citrullination in rhG1 were explored by molecular modeling. CitPG-specific serum autoantibodies were quantified by enzyme-linked immunosorbent assays, and CitPG was localized in osteoarthritic (OA) and RA cartilage using immunohistochemistry.

**Findings:**

Sera from ACPA+ RA patients reacted with PG purified from normal human cartilage specimens. PG fragments (mainly those containing the G1 domain) from OA or RA cartilage extracts were recognized by ACPA+ sera but not by serum from ACPA- individuals. ACPA+ sera also reacted with *in vitro* citrullinated rhG1 and G3 domain-containing fragment(s) of PG. Molecular modeling suggested multiple sites of potential citrullination within the G1 domain. The immunohistochemical localization of CitPG was different in OA and RA cartilage.

**Conclusions:**

CitPG is a new member of citrullinated proteins identified in human joints. CitPG could be found in both normal and diseased cartilage specimens. Antibodies against CitPG may trigger or augment arthritis by forming immune complexes with this autoantigen in the joints of ACPA+ RA patients.

## Introduction

Rheumatoid arthritis (RA) is an autoimmune disease of the synovial joints causing chronic inflammation and profound tissue destruction in affected patients. The pathological features of RA include infiltration of the joints by inflammatory cells and formation of invasive synovial pannus, ultimately resulting in cartilage and bone erosion and loss of joint function [1][2]. The autoimmune character of RA is underscored by prominent production of autoantibodies (autoAbs) such as those against IgG (rheumatoid factor, RF), and a broad array of joint tissue-specific and other endogenous citrullinated proteins [3][4][5].

Citrullination is a post-translational protein modification catalyzed by peptidyl arginine deiminase (PAD) enzymes, resulting in the conversion of protein-bound arginine to citrulline. Among PAD enzymes, PAD4 has been implicated in physiological processes such as the normal regulation of gene expression via citrullination of histones as well as in autoimmunity by generating autoantigens (neoepitopes) through citrullination of self-proteins in RA [6][7]. Anti-citrullinated protein Abs (ACPA) can be detected in the serum of an even higher proportion of RA patients than RF [3][4][8], and ACPA positivity is employed as a diagnostic and prognostic tool for this disease [4][8][9][10].

The serum ACPA-reactive proteins identified thus far include citrullinated filaggrin, fibrinogen, vimentin, type II collagen (CII), α-enolase, and a few viral antigens (reviewed in [5] [6][7][8][10]). Previous studies have described T-cell reactivity with citrullinated proteoglycan (PG) aggrecan peptides in RA patients [11][12][13] and one group reported the presence of PG G1 domain-specific autoAbs in RA synovial fluid (SF) [14]. However, PG-specific ACPA have not been described, and it is not known if cartilage PG undergoes citrullination in vivo.

Citrullinated proteins and PAD4 enzyme have been identified in rheumatoid synovial tissue [15][16]. In addition, elevated concentrations of ACPA in the SF relative to the serum level in the same RA patients suggest that SF ACPA (reactive with multiple citrullinated proteins) might be preferentially retained or locally produced in the joint [17][18][19]. The citrullinated proteins within joint tissues provide obvious targets for ACPA, leading to immune complex formation [20]. As complement-fixing Abs/immune complexes can trigger inflammatory cell recruitment [6][21], ACPA have a significant potential to initiate inflammation or amplify the inflammatory cascade in the RA joint.

We found high ACPA levels in the sera of mice immunized with cartilage PG (PG-induced arthritis, PGIA) [22], but not in non-immunized BALB/c mice or in those immunized with human CII. As these mice were injected with human cartilage PG aggrecan (henceforth PG) emulsified in a synthetic (protein-free) adjuvant, we suspected that the PG used for immunization had already contained citrullinated molecules. Thus, we sought to detect citrulline in PG isolated and purified from normal articular cartilage specimens. Indeed, we found that some samples of PG purified from normal adult cartilage contained citrulline residues, as the sera from ACPA+ RA patients reacted with these PG samples. Moreover, we could identify citrullinated (ACPA-reactive) PG epitopes in crude extracts and tissue sections of cartilage obtained from osteoarthritic (OA) and RA joints and most of the citrulline residues were located in the G1 domain of the core protein of cartilage PG aggrecan.

## Materials and Methods

### Human cartilage and serum samples

PG aggrecan molecules (PG monomers) were isolated from normal human cartilage and purified by repeated cesium chloride (CsCl) gradient ultracentrifugation as previously described [23][24]. We used “archived” samples of PG monomers purified from knee cartilage of adult organ donors (with no history of joint disease and having macroscopically normal-looking cartilage). CII was also isolated from normal human cartilage by limited pepsin digestion and NaCl precipitation as described [25]. Purified PG and CII were stored at -80°C under nitrogen.

Cartilage tissue was obtained from patients undergoing knee joint replacement surgery. Written informed consent was obtained from each patient before their participation, and cartilage specimens were provided through the Orthopedic Information, Tissue and Implant Repository Study approved by the Institutional Review Board (IRB) of Rush University Medical Center Chicago, IL (IRB approval number L00011021). These knee cartilage samples were pulverized in liquid nitrogen and crude extracts were prepared with 4.0 M guanidine hydrochloride in the presence of protease inhibitors [26][27]. The samples were dialyzed, analyzed for PG content [26][27] and stored at -80°C.

Peripheral blood was obtained from RA patients (84 ACPA-positive and 20 ACPA-negative) and 16 healthy volunteers at Rheumatology Associates, Rush University Medical Center, and at the Department of Rheumatology, Faculty of Medicine, University of Debrecen, Hungary. All subjects provided their written informed consent to participate in this study. Collection of blood from the study participants at these two rheumatology clinics was approved by the IRB of Rush University Medical Center (approval number L89050101) and the Ethics Committee of the Faculty of Medicine, University of Debrecen (approval number 29643/2012/EKU), respectively. All RA patients fulfilled the ACR 1987 RA classification criteria [28], 84 of them were positive for serum ACPA (>25 U/ml) and had a disease duration of 10.56 ± 0.88 years (mean ± SEM). None of the RA patients received B-cell depletion therapy such as Rituximab.

### Assaying ACPA levels in human serum

The ACPA levels in serum samples of the 84 ACPA+ and 20 ACPA- RA patients (and healthy controls) were re-assessed simultaneously using enzyme-linked immunosorbent assay (ELISA) kits for anti-mutated citrullinated vimentin (MCV, Antibodies-online, Inc., Atlanta, GA) and anti-citrullinated cyclic peptide (CCP3 kit, Inova Diagnostics, San Diego, CA). The sera were assayed at 1:100 and 1:500 dilutions, and the assays were performed according to the manufacturers’ instructions. We selected 2 RA serum samples (ACPA+#9 and ACPA+#20) with high anti-MCV and anti-CCP3 titers (both ~1100 U/ml) to detect citrullinated proteins by dot blot, Western blot, and immunohistochemistry. As an additional positive control for dot blot, we also used the “Calibrator A” component, a serum standard (pre-diluted to 250 U/ml) supplied with the anti-CCP3 kit. ACPA-negative (<20 U/ml) normal human serum samples served as negative controls in different immunoassays.

### Dot blot, gel electrophoresis and Western blot

Dot blots were performed using a 96-well transfer system with nitrocellulose membranes (Bio-Rad, Hercules, CA). Dots of purified PG, CII or crude cartilage extracts as well as serum IgG controls were applied to the membranes. Dotted membrane strips were probed with ACPA+ or ACPA- human serum (1:2,000 dilution), followed by horseradish peroxidase (HRP)-conjugated anti-human IgG (1:5,000 dilution). Control probes included anti-IgG Abs, or PG- or CII-specific monoclonal Abs (mAbs) (1:5,000–1:10,000 dilutions). For electrophoresis, samples (30 μg protein/lane) were loaded onto 8% sodium dodecyl-sulfate polyacrylamide gels (SDS-PAGE), and stained with 0.1% toluidine blue (for glycosaminoglycan, GAG) or Coomassie blue (for proteins). To facilitate the entry of macromolecules into the gels and subsequent resolution of protein bands, the chondroitin sulfate (CS) GAG side chains of PG were removed (truncated) by digestion with chondroitinase ABC (Seikagaku America, East Falmouth, MA) as previously described [23]. Equal amounts of protein (5 μg/lane) of the deglycosylated OA cartilage extract were loaded onto 8% SDS-PAGE and transferred onto nitrocellulose membrane. Vertical strips of the membrane were probed with ACPA+ or ACPA- human serum, or with mAbs to hPG G1- (G18) [22] or G3- (LEC-7, Acris Antibodies, San Diego, CA) domain, mAbs to hCII and C4S (BE123, Chemicon International Temecula, CA), or with rabbit Abs to enzyme-generated -VDIPEN and -NITEGE neoepitopes [29]. The Ab-stained dots or bands were visualized with HRP-labeled second-step antibodies and enhanced chemiluminescence (Luminol enhancer solution, Amersham/GE Healthcare Life Sciences, Piscataway, NJ).

### Removal of contaminating serum IgG from the cartilage extracts and immunodepletion of the G1 domain

While the highly purified PG samples were free of serum proteins, the crude extracts of human cartilage specimens were contaminated with human serum components. Therefore, it was necessary to remove serum IgG contamination from the cartilage extracts prior to using them for dot blot or Western blot. The crude extracts were first incubated with purified goat anti-human IgG (Invitrogen). The IgG-anti-IgG immune complexes were then removed by repeated incubation with Protein G-Sepharose CL4B until the HRP-conjugated goat anti-human IgG antibody no longer stained the cartilage extract on the membrane. A similar method was employed to deplete the G1 domain-containing PG fragments in the crude extracts by immune absorption using the anti-G1 mAb (G18) followed by incubation with Protein G-Sepharose CL4B.

### Immunohistochemistry

Cryostat sections of knee cartilage (tibial plateaus of OA and RA patients) were fixed in cold methanol for 5 min, blocked with 1% normal goat serum at room temperature for 1 hour, and incubated with ACPA+ or ACPA- human sera at 1:50–1:100 dilutions. After extensive washing in phosphate-buffered saline (PBS; pH 7.4), sections were incubated with Texas Red (TR)-conjugated goat anti-human IgG or biotinylated anti-human G1 mAb (G18) followed by Alexa Fluor (AF)488-labeled streptavidin, and mounted in DAPI-containing Vectashield^R^ Hard Set^TM^ mounting medium (Vector Laboratories, Burlingame, CA). ACPA- sera or non-biotinylated G18 mAb and fluorochrome-labeled reagents (without primary Abs) were used as controls. Fluorescence staining was examined and images were created using a Nikon FXA epifluorescence microscope (Nikon, Melville, NY) equipped with a digital color camera and MetaMorph image processing program (Meta Imaging Series, Universal Imaging Corporation, Downingtown, PA).

### *In vitro* citrullination of the rhG1 domain and human CII, and detection of anti-CitPG and anti-CitCII Abs by ELISA

Purified rhG1 protein (2.5 mg) was dissolved in citrullination buffer (0.1 M Tris-HCl [pH 7.6] containing 10 mM CaCl_2_ and 5 mM dithiothreitol) [30]. Lyophilized human CII [25] was first dissolved in 0.1 M acetic acid (5 mg/ml) and dialyzed exhaustively against the citrullination buffer. Proteins were incubated with rabbit skeletal muscle PAD4 (Sigma-Aldrich), at a concentration of 4 U/mg protein for 4 hours at 37°C. Citrullination was terminated by addition of 20 mM EDTA and the material was dialyzed successively against 10 mM Tris-HCl buffer containing 5 mM EDTA (pH 7.6) at 4°C. Control (non-citrullinated) rhG1 and CII proteins were treated identically, except that no PAD4 was added.

The effect of PAD4 treatment (citrullination status) on rhG1 and CII was detected using ACPA+ serum or ACPA-independent methods. In brief, untreated and PAD4-treated proteins were labeled with rhodamine-conjugated phenylglyoxal, a citrulline-specific probe (Cayman Chemical, Ann Arbor, Michigan), according to a protocol described in [31] before SDS-PAGE, and fluorescent bands were detected with a Bio-Rad ChemiDoc imaging system (Bio-Rad, Hercules, CA). Another method of citrullinated protein detection involved the use of an anti-modified citrulline detection kit, following the manufacturer’s instructions (EMD Millipore, Billerica, MA) [32].

The optimal coating concentrations for ELISA were 0.2 μg CitPG or rhG1 (non-CitPG), and 0.5 μg CitCII or non-citrullinated CII per well. Sera from RA patients were diluted to 1:100 and 1:500, and 100 μl of the serum samples were incubated with the immobilized antigens at room temperature for 1 hour, followed by washing and incubation with HRP-conjugated goat anti-human IgG for 1 hour. The color reaction was developed using 3,3',5,5'-tetramethylbenzidine (TMB) substrate (BD Biosciences, San Diego, CA). Optical density (OD) values (at 450 nm) of non-citrullinated proteins (PG and CII) were subtracted from those of CitPG or CitCII, respectively. The specific reactivity of the sera with CitPG or CitCII was expressed as ΔOD.

### Three-dimensional (3-D) molecular modeling of the G1 domain of human PG

The theoretical structure of the G1 domain was assembled via protein homology modeling using the SWISS-MODEL Workspace program (http://www.swissmodel.expasy.org). The molecular coordinate files of the Aloop, Bloop and B’loop of the G1 domain were created based on the SWISS-MODEL repository templates 1qz1A, 1o7bT, and 1pozA, respectively [33][34]. The 3D structures of the protein were visualized and the arginine residues were highlighted using RasMol software (http://www.RasMol.org) [35].

### Statistical analysis

The statistical analysis of data was performed using GraphPad Prism 6 program (GraphPad Software, La Jolla, CA). Pearson’s test was employed to determine the correlation coefficients (r), and the best-fit curves were created using linear regression. P values of <0.05 were considered significant.

## Results

### CitPG (Cit-Aggrecan) is detected in normal adult human cartilage by ACPA+ sera

**Fig 1:**
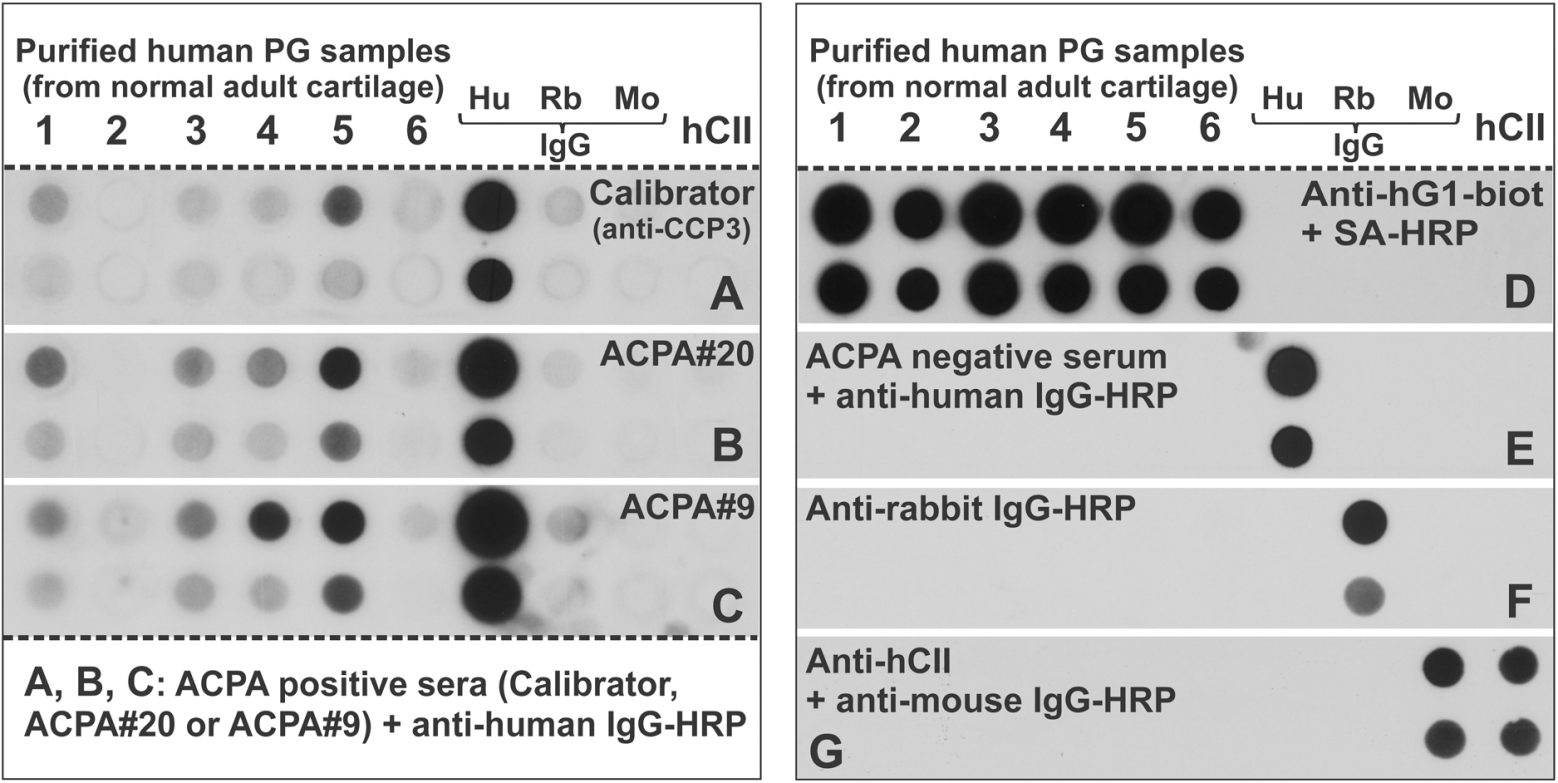
Recognition of proteoglycan (PG) aggrecan purified from normal human cartilage by ACPA-positive human sera. (**A-C**) Dots of human PG aggrecan that was purified from 6 normal cartilage samples (1–6) were applied to nitrocellulose membranes (upper dots: 2 μg, lower dots: 0.2 μg PG) along with various control IgGs purified from human (Hu), rabbit (Rb) or mouse (Mo) serum (upper dots: 20 ng, lower dots: 2 ng IgG) and human type II collagen (hCII) purified from normal cartilage (upper dot: 10 μg, lower dot: 1 μg hCII). The membranes were subjected to immunostaining with ACPA-positive sera including the (**A**) “Calibrator” serum from the anti-CCP3 assay kit and (**B, C**) sera from two ACPA+ RA patients (#20 and #9), followed by horseradish peroxidase (HRP)-labeled anti-human IgG. (**D-G**) Specificity controls included blotting with (**D**) a biotinylated monoclonal antibody (mAb) specific to human PG G1 domain (anti-hG1-biot) followed by streptavidin (SA)-HRP, (**E**) ACPA- serum followed by anti-human IgG-HRP, (**F**) anti-rabbit IgG-HRP, and (**G**) mouse antibody to hCII (anti-hCII) followed by anti-mouse IgG-HRP.

The intact PG monomers in normal cartilage are very large molecules (~ 2x10^6^ Da) and cannot be resolved in SDS-PAGE. Therefore, we applied dots of highly purified PG aggrecan molecules (collected from 6 normal adult cartilage samples, age range 26-76-years) [23][24] along with control samples including purified human, rabbit, and mouse IgGs as well as hCII to a nitrocellulose membrane (Fig 1). The calibrator serum from CCP3 ELISA kit reacted with 2 of the 6 PG dots (Fig 1A), whereas both ACPA+#20 and ACPA+#9 sera recognized 4 of the 6 PG samples (Fig 1B and 1C). None of the ACPA+ sera reacted with hCII, and the secondary Ab was positive only with human IgG (Figs 1A–1C). When the membrane was probed with the hG1-specific mAb G18 [22], all of the PG samples, but not the control IgGs or hCII, were recognized by this mAb (Fig 1D). Importantly, an ACPA- serum did not react with any of the PG or other control dots. Specificity controls (human, mouse, rabbit IgGs) used in subsequent experiments reacted only with the specific antibodies (Fig 1D–1G).

### CitPG is also present in cartilage extracts from OA and RA patients

**Fig 2:**
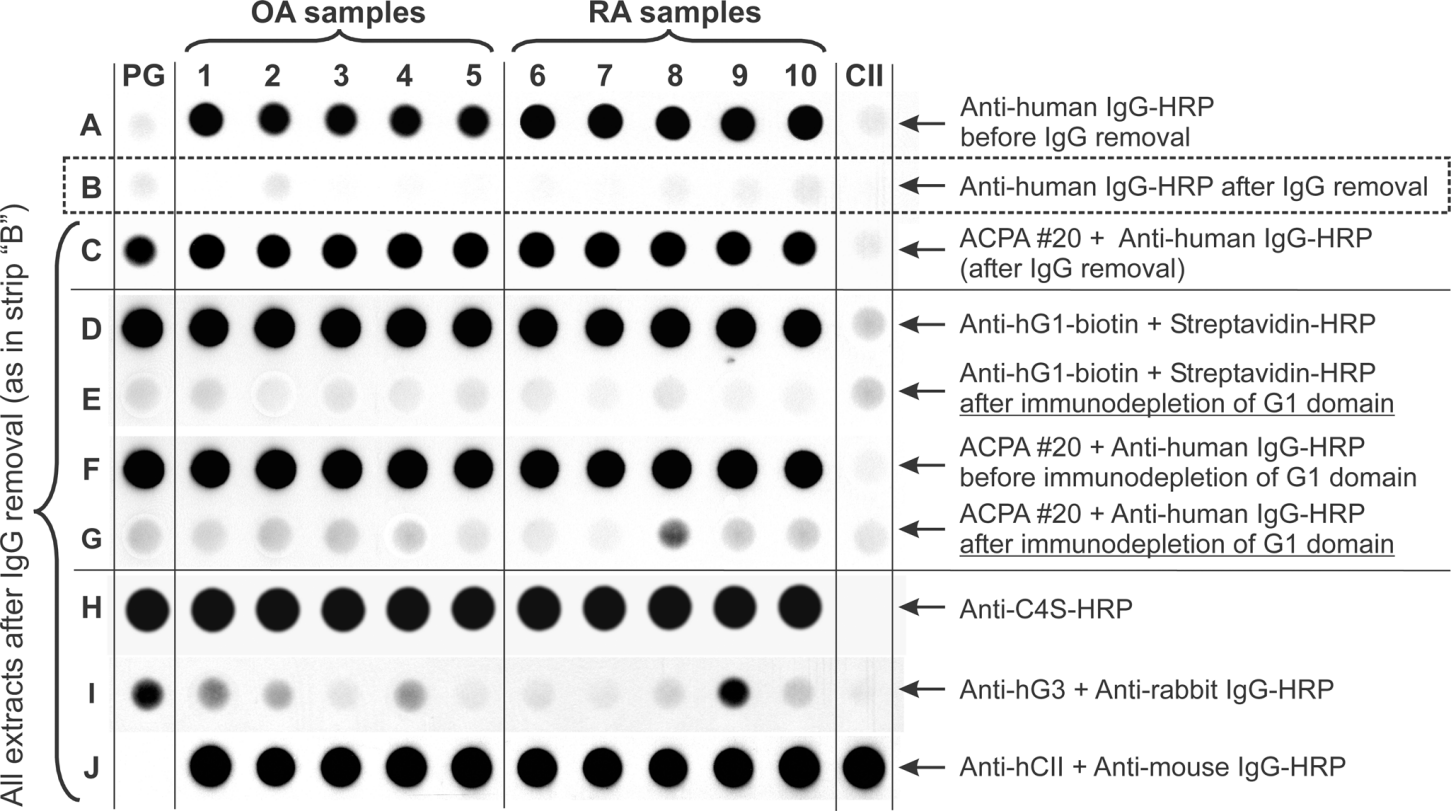
Recognition of PG in crude extracts of osteoarthritis (OA) and rheumatoid arthritis (RA) cartilage specimens by ACPA and PG-specific antibodies. Dots of crude extracts of cartilage from OA donors (row A, samples 1–5) and RA donors (samples 6–10) were applied to a nitrocellulose membrane strip. Purified human PG and CII (far left and far right dots, respectively) served as controls. Blotting with the secondary antibody (anti-human IgG-HRP) revealed positive reactions (row A) with all crude extracts, but not with purified hPG or hCII. The “positive” reaction disappeared after the contaminating IgG was removed from the crude extracts (row B). These IgG-free cartilage extracts were used for subsequent dot blots. The ACPA+#20 serum (row A) reacted with all cartilage extracts and purified PG (row C). Similarly, the G1 domain-specific mAb recognized purified PG and PG in the crude extracts (row D), but no reaction was detected when the G1 domain was removed by immune absorption (row E). The reactivity of the cartilage extracts with ACPA+#20 serum (row F) was nearly completely lost after G1 domain immunodepletion (row G). As demonstrated by the anti-chondroitin 4-sulfate (C4S)-specific antibody, PG in all extracts and in the purified PG sample were glycosylated (row H). Some extracts and the purified PG contained small amounts of the PG G3 domain (row I), and all crude extracts contained cartilage-specific CII (row J).

We hypothesized that if PG citrullination occurs in macroscopically intact (normal) human cartilage (Fig 1), it should also occur in joint diseases such, as OA or RA. As noted above, due to its large size, the intact cartilage PG aggrecan molecule cannot be resolved by SDS-PAGE, unless the GAG side chains are removed or the core protein is degraded, or both. Although limited core protein degradation occurs during the normal turnover of PG, the core protein is degraded extensively in diseased OA or even more in RA cartilage [36]. Unlike intact PG from normal cartilage, PG fragments present in OA or RA cartilage cannot be separated or purified from other proteins by CsCl gradient ultracentrifugation. Therefore, we used crude extracts of RA and OA knee cartilage specimens (without any purification) to identify citrullinated PG by dot blots. However, these crude extracts were contaminated with human serum proteins including IgG, as the dots of all cartilage extracts gave positive reaction with anti-human IgG-HRP Ab (Fig 2, top row A). The crude cartilage extracts of OA and RA patients were subjected to immune absorption with goat anti-human IgG-Protein G-Sepharose, which resulted in the removal of serum IgG contamination (Fig 2, row B). All 10 pre-absorbed extracts (and purified human PG, but not purified human CII) reacted with the ACPA+ serum (Fig 2. row C). Importantly, all samples (and purified PG) also reacted with the G18 anti-hG1 mAb (Fig 2, row D). The reaction was specific, as the G18 mAb did not react after G1 depletion (Fig 2, row E). The ACPA+ RA serum stained the purified human PG dots as well as all of the OA and RA cartilage extracts, suggesting that all these samples contained citrullinated PG molecules (Fig 2, row F). This positive staining with the ACPA+ serum disappeared after removal of the G1 domain by immune absorption (Fig 2, row G) indicating that most, if not all, of the citrulline residues were in the N-terminal G1 domain of PG or G1 domain-containing PG fragments. The PG molecules in all tested samples had chondroitin sulfate side chains or stubs as indicated by positive reactions with an anti-C4S mAb (Fig 2, row H). A few samples also contained the C-terminal G3 domain of PG (Fig 2, row I). Finally, all crude cartilage extracts, but not purified PG, contained CII, as they gave positive reaction with the anti-hCII mAb (Fig 2, row J). Collectively, these data suggested that crude extracts of OA and RA cartilage specimens contained citrullinated PG, and that the majority of the citrulline residues were located within the G1 domain of the PG molecule.

**Fig 3:**
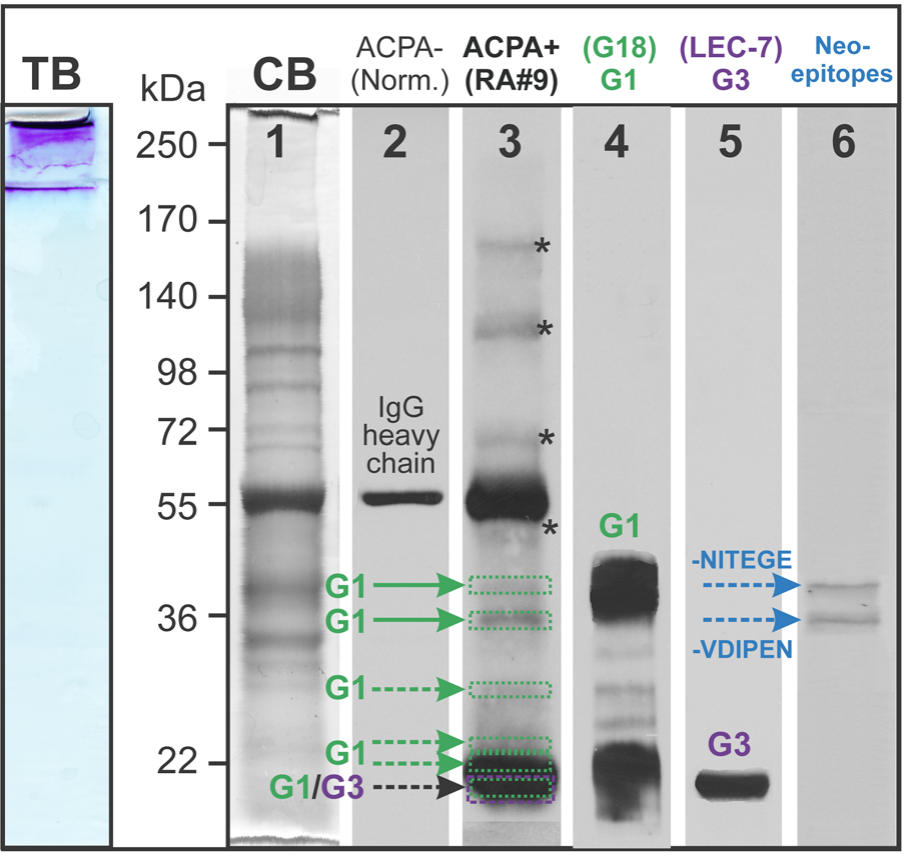
Western blots to identify PG domains and fragments of OA cartilage extract recognized by ACPA-positive serum. The crude extract of OA cartilage (shown as sample 4 in Fig 2) was loaded onto 8% SGS-PAGE gel and stained with toluidine blue (TB) to visualize PG GAG chains. Essentially all of the TB-positive PG material remained in the stacking gel. To facilitate resolution, chondroitin sulfate chains were removed by digestion with chondroitinase ABC, and aliquots of the deglycosylated extract was loaded onto 6 lanes of a SDS-PAGE gel. Coomassie blue (CB) staining of the gel (lane 1) showed good resolution of the proteins of the OA cartilage extract after deglycosylation. Following transfer onto a nitrocellulose membrane, vertical strips of the membrane were probed with human sera or PG-specific antibodies (lanes 2–6). Immunostaining with ACPA- (normal) serum followed by anti-human IgG-HRP revealed a single protein band most likely corresponding to the heavy chain of contaminating IgG (lane 2). The ACPA+ serum detected several additional bands (lane 3). To identify these bands, replicate strips of the membrane were probed with antibodies against the G1 or G3 domain of PG and a pair of antibodies recognizing protease-generated PG neoepitopes. The respective antibodies showed reactions with the G1 (lane 4), G3 (lane 5) as well as with the neoepitopes -NITEGE and -VDIPEN (lane 6). There were additional bands above 55 kDa (depicted with asterisks in lane 3) that could not be identified as PG fragments. One representative sample of over 10 Western blots (using different crude extracts and ACPA+ sera) is shown.

To corroborate these results, we sought to identify citrullinated G1 domain (and perhaps other citrullinated fragments) of the PG molecule by SDS-PAGE and Western blotting. However, PG fragments in crude extracts of OA cartilage were still too large to enter the running gel (Fig 3, lane “TB”: toluidine blue-stained for GAG content). Deglycosylation of the PG (removal of GAG chains) of the OA cartilage extract provided good resolution on SDS-PAGE (Fig 3, lane “CB”: Coomassie blue-stained for proteins). Aliquots of this OA extract were loaded onto SDS-PAGE gels and transferred to nitrocellulose membranes. As shown in the other lanes of Fig 3, the normal (ACPA-) human serum did not stain the OA extract, and only some residual contaminating IgG was recognized by the secondary anti-human IgG Ab (lane 2). In contrast, several protein bands were observed after blotting with the ACPA+ RA serum (lane 3). Using PG domain- and neoepitope-specific antibodies, the bands recognized by the ACPA+ serum (lane 3) could be identified as citrullinated PG fragments containing the G1 domain (lane 4), the G3 domain (lane 5) and G1-associated fragments with protease cleavage sites (lane 6). Four of the bands stained with the ACPA+ serum (lane 3, depicted by *) could not be identified as either G1-, interglobular- or G3 domain-containing fragments.

### Immunolocalization of CitPG epitopes in tissue sections from OA and RA cartilage specimens

**Fig 4:**
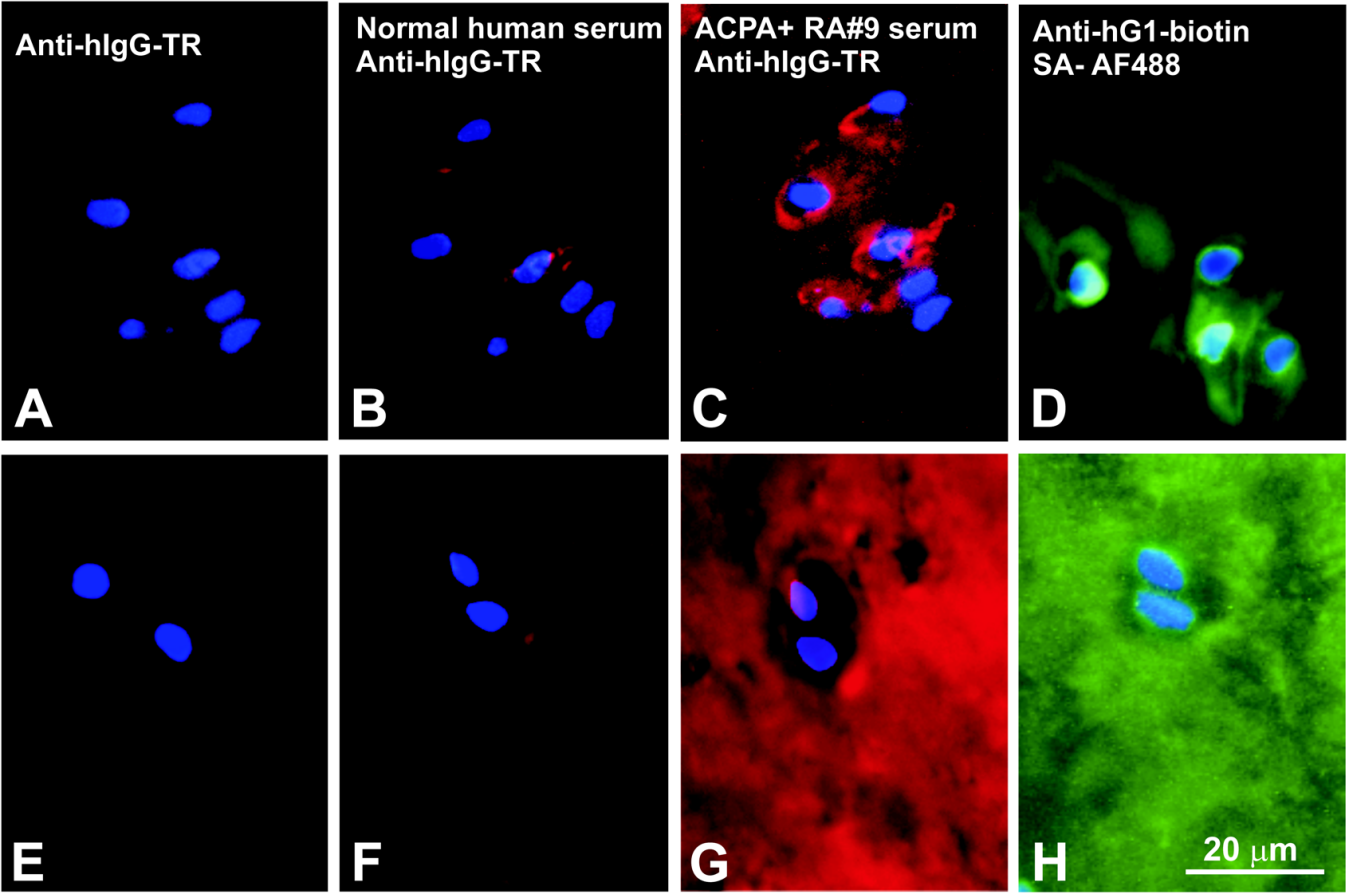
Immunohistochemical localization of ACPA-reactive (citrullinated) epitopes in OA and RA cartilage sections. (**A-D**) Frozen sections of OA knee (tibial plateau) cartilage were immunostained with (**A**) Texas red-labeled anti-human IgG (anti-hIgG-TR), (**B**) normal (ACPA-negative) human serum followed by anti-hIgG-TR, (**C**) ACPA+ serum (RA#9) followed by anti-hIgG-TR, or (**D**) a biotinylated anti-human G1 antibody followed by Alexa Fluor 488-labeled streptavidin (SA-AF488). (**E-H**) Frozen sections prepared from the RA tibial plateau cartilage were immunostained with the same sera and antibodies as listed for **A-D**. Cell nuclei in all sections were visualized by DAPI staining. (**A** and **E**) Anti-hIgG-TR alone did not stain the sections, and (**B** and **F**) negligible reaction (red fluorescence) was observed when the tissues were first stained with ACPA- serum. (**C**) The ACPA+ serum primarily stained the chondrocyte pericellular matrix in the OA cartilage, but (**G**) it diffusely stained the entire matrix of the RA cartilage. Similar staining patters to those with ACPA+ serum were observed when (**D**) the OA and (**H**) RA cartilage sections were incubated with biotinylated anti-hG1 mAb (green fluorescence), suggesting at least partial co-localization of PG G1 and citrullinated epitopes in both OA and RA cartilage.

Incubation of OA and RA cartilage sections with anti-human IgG-Texas Red (anti-hIgG-TR, Fig 4A and 4E) or with normal (ACPA-) human serum, followed by anti-hIgG-TR (Fig 4B and 4F) resulted in some background staining, likely due to the presence of trace amounts of human IgG in these cartilage specimens. When ACPA+ human serum was used as a source of primary Ab, strong positive staining was observed in both the OA and RA cartilage sections (Fig 4C and 4G). ACPA positivity was primarily observed in the intra- and pericellular areas of the OA cartilage (Fig 4C), whereas it was detected throughout the entire matrix in the RA cartilage (Fig 4G). Immunohistochemistry on neighboring sections of the same OA and RA specimens with G18 (anti-hG1) mAb resulted in staining patterns (Fig 4D and 4H) similar to those obtained with the ACPA+ serum (Fig 4C and 4G).

### Potential sites of citrullination within the G1 domain of human PG and detection of citrullinated rhG1 by ACPA and other probes

**Fig 5:**
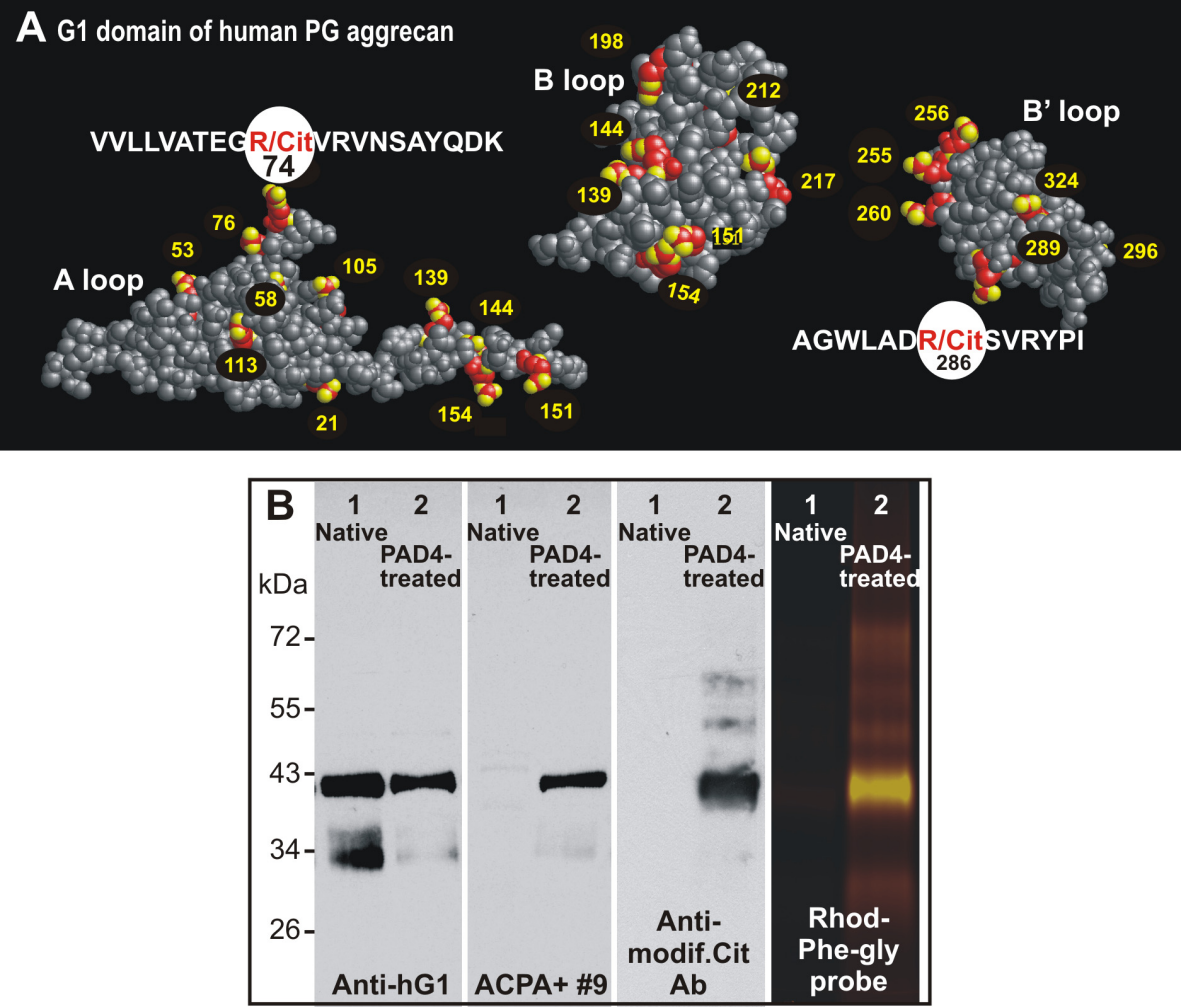
Positions of arginine residues within the three-dimensional structure of the human G1 domain and detection of citrullinated rhG1. (**A**) 3-D images of the A, B, and B’ loops of the G1 domain illustrate the location of arginine (R) residues (red-yellow balls with numbers) and the two previously reported immunodominant epitopes (sequences highlighted), both of which contain arginine residues that may become citrulline (R/Cit) [11][12][13]. The three loops of G1 were rotated relative to each other using RasMol software in order to expose the R-rich surfaces. The amino acid sequence is shown in S1 Fig. (**B**) *In vitro* citrullination of rhG1 was performed using PAD4 enzyme. The native (lanes 1) and PAD4-treated (lanes 2) rhG1 proteins were loaded onto SDS-PAGE gels and transferred to nitrocellulose membranes. Two of the membranes were probed with either anti-hG1 mAb: (first panel) or with ACPA+ RA serum (second panel). The citrulline residues present in the same proteins on the third membrane were subjected to chemical modification and then probed with an Ab specific for chemically-modified citrulline (anti-modif. Cit Ab, third panel). Native and PAD4-treated rhG1 proteins were also reacted with citrulline-specific phenylglyoxal conjugated with rhodamine (Rhod-Phe-Gly) and subjected to SDS-PAGE (fourth panel). While the anti-hG1 mAb reacted with both the native and citrullinated forms of the protein, only the citrullinated hG1 was detected by ACPA, the anti-modified citrulline Ab, and the phenylglyoxal probe.

The G1 domain of human PG contains 23 arginine residues (S1 Fig) that can be potentially converted to citrulline by PAD enzymes. Molecular modeling revealed that most arginine residues were displayed on the surfaces of the three subdomains/loops (A, B, and B’) of the G1 domain, theoretically accessible to PADs (Fig 5A). The localization of recently identified T-cell epitopes within the A and B’ loops of the G1 domain is highlighted in Fig 5A.

To test the specificity of the PG/G1-reactive ACPA+ RA serum (ACPA+#9) against the citrullinated form of PG, we treated the rhG1 domain of PG [22] with PAD4 enzyme, and then subjected these native and citrullinated rhG1 proteins to Western blotting with anti-hG1 mAb, ACPA+#9 serum, and an Ab to chemically modified citrulline. As an Ab-independent method, we also used a rhodamine-labeled phelylglyoxal probe that specifically reacts with citrulline [31]. While the anti-hG1 mAb reacted with both the native and citrullinated form of rhG1 (Fig 5B, first panel), only the citrullinated protein was recognized by the ACPA+ serum (Fig 5B, second panel). Citrullination of rhG1 (and the specificity of ACPA) was further confirmed by an ACPA-independent method using an Ab against modified citrulline (Fig 5B, third panel) and by a chemical method using the citrulline-specific phenylglyoxal probe (Fig 5B, fourth panel).

### Using CitrhG1 to detect PG-specific ACPA in the sera of RA patients

**Fig 6:**
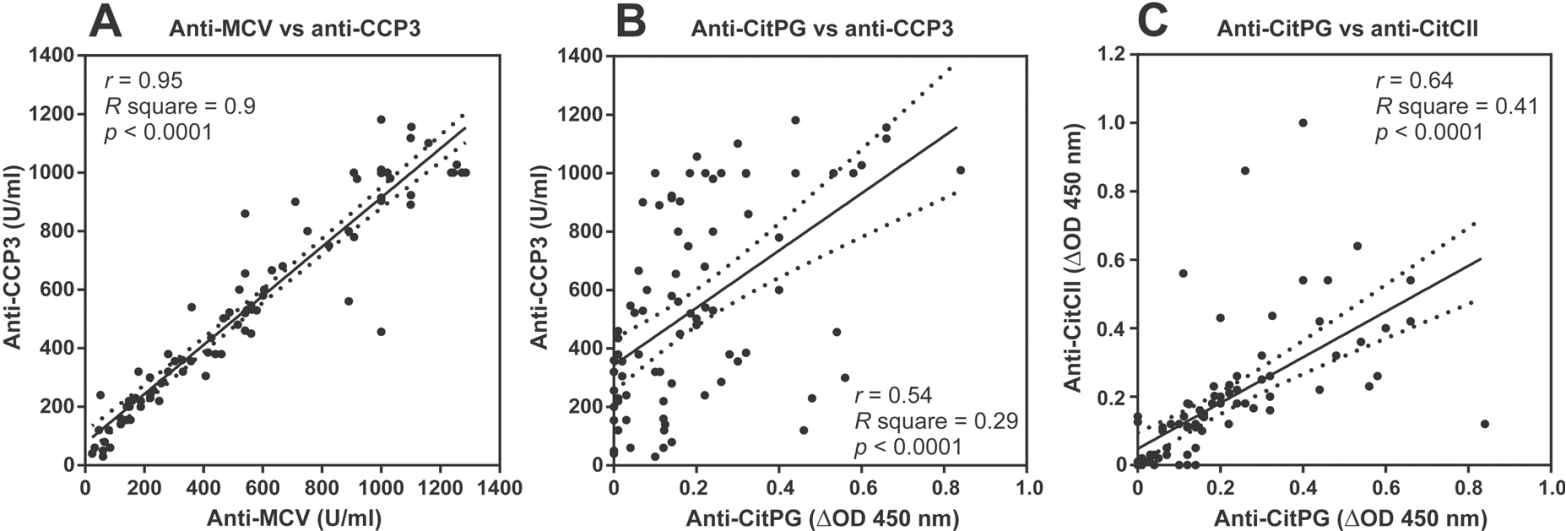
Correlations between ACPA of different specificities including citrullinated PG in the sera of RA patients. (**A**) Concentrations of antibodies to mutated citrullinated vimentin (MCV) and cyclic citrullinated peptides (CCP) were measured in serum samples from 84 RA patients using commercial ELISA kits. Results were expressed as units/ml (U/ml). Correlation analysis revealed strong positive correlation between anti-MCV and anti-CCP3 levels (r = 0.95, R square = 0.9, p<0.0001). (**B**) Concentrations of anti-CitPG antibodies were measured by in-house ELISA using *in vitro* citrullinated rhG1 (shown in Fig 5B) as antigen (CitPG). The results were expressed as delta optical density (ΔOD, the OD values of anti-CitPG minus the OD values of anti-PG as described in the Methods). The ΔOD values were correlated with the concentrations (U/ml) of anti-CCP in the same 84 RA serum samples. (**C**) Purified hCII was also citrullinated by PAD4, and CitCII was used to measure anti-CitCII levels in the 84 RA serum samples by ELISA. The dotted lines indicate the 95% confidence intervals.

We sought to determine the relative proportions of citrullinated PG (CitrhG1/CitPG)-specific and citrullinated CII (CitCII)-specific ACPA in our collection of 84 RA serum samples (all of which were previously found to be ACPA+). We assayed these samples simultaneously using commercially available ELISA kits for anti-MCV and anti-CCP3, and in-house ELISAs employing PAD4-treated rhG1 (CitPG) and PAD4-treated human CII (CitCII). As seen in Fig 6A, all 84 of the previously tested ACPA+ sera had comparable reactivity with MCV (range: 12–1285 U/ml) and CCP3 (range: 10–1160 U/ml), showing a strong positive correlation (*r* = 0.95, *p* < 0.0001). Of note, a few RA samples contained less than 20 U/ml ACPA (the cut-off value for ACPA positivity) in one or both of the ACPA assays. Of the same 84 RA serum samples, 61 (72.6%) reacted with CitPG, 51 (60.7%) reacted with both CitPG and CitCII, and only 23 (27.4%) of the CCP3+ sera were negative for CitPG and/or CitCII (Fig 6B and 6C). There was relatively poor, but significant, correlation (*r* = 0.54, *p* < 0.0001) between the anti-CitPG and anti-CCP3 levels (Fig 6B), whereas the anti-CitPG and anti-CitCII ΔOD values showed better correlation (*r* = 0.64, *p* < 0.0001) (Fig 6C).

## Discussion

In this study we demonstrate that ACPA+ sera from RA patients react with human cartilage PG aggrecan. Our investigations were prompted by the observation that immunization of BALB/c mice with purified PG [37] or crude extracts of OA cartilage [26] induced both arthritis and abundant production of ACPA [22]. Indeed, we found that ACPA of RA patients reacted with PG fragments of OA and RA cartilage, suggesting that cartilage from diseased joints contained in vivo citrullinated PG molecules. Unexpectedly, ACPA of RA patients also reacted with highly purified PG monomers isolated from visually normal cartilage specimens of adult human subjects. The significance of our observations is three-fold. First, PG can be citrullinated in vivo in the joints of adult human subjects; second, CitPG-specific Abs are present in the serum of ACPA+ RA patients; and third, CitPG epitopes may also induce ACPA production in patients with RA.

Removal of the G1 domain of human PG by immunodepletion resulted in substantial weakening of reactivity indicating a relatively high concentration of citrullinated epitopes located within the G1 region. The 325 amino acid-long G1 domain contains 23 arginine residues, and the G2 and G3 domains contain 13 and 20 arginines, respectively. The linear GAG-attachment domains have only 11 arginine residues. While all 67 arginine residues of PG (56 of which are found in the globular domains) are potential sites of in vivo citrullination by PAD enzymes, steric hindrance by GAG side chains and PG-bound hyaluronan may limit the access of PAD to these sites either within the GAG-attachment domains or in the B loop of the G1 domain [38][39]. Here, we show that ACPA from RA serum preferentially react with G1- and G3-containing PG fragments. Identification of citrulline residues within the G1 and G3 domains of human PG and detection of ACPA that preferentially bind to citrulline-containing epitopes (peptides) within these regions, are the subjects of our ongoing investigations.

We were able to detect ACPA-reactive epitopes within OA and RA cartilage specimens by immunohistochemistry. While ACPA-reactive regions were restricted to the intra- and pericellular compartments of OA cartilage, ACPA reactivity was distributed throughout the entire matrix of RA cartilage. The similar immunolocalization of PG (G1)-specific and ACPA-reactive regions suggested that at least some of the citrullinated epitopes recognized by ACPA are CitPG epitopes in both OA and RA cartilage sections. The wide distribution of citrullinated epitopes within RA cartilage suggests that PADs released from inflammatory cells in synovial fluid or from synovial tissue may gain access to the extensively damaged cartilage matrix in RA joints. In contrast, the intra- and pericellular localization of the citrullinated epitopes in OA cartilage suggests that chondrocyte-derived PADs may contribute to the citrullination of matrix molecules, including PG. Further studies are warranted to determine if PADs produced by chondrocytes are involved in the citrullination of human cartilage matrix molecules.

A human PG peptide representing the so-called 5/4E8 epitope (ATEGRVRVNSAYQDK) was found by our group to be a dominant T-cell epitope in PG-immunized arthritic BALB/c mice [38][40][41]. This epitope was also the target of in vitro studies in two independent laboratories [11][12] showing that a citrullinated and extended version of the 5/4E8 epitope-containing synthetic peptide (VVLLVATEG**Cit**VRVNSAYQDK) elicited strong T-cell responses in RA patients. This citrullinated peptide induced substantial cytokine (IL-17, IL-22, IL-6, TNFα, and IFNγ) production by peripheral blood T cells from the majority of RA patients [11][12]. T cells from RA patients responded poorly to the native (non-citrullinated) peptide in both studies, and T cells from healthy subjects did not respond at all [11] or responded only to the citrullinated peptide by producing IL-6 [12]. More recently, Aggarwal *et al*. [13] reported that another citrullinated peptide (AGWLAD**Cit**SVRYPI) from human PG induced as much T-cell proliferation as citrullinated vimentin or fibrinogen peptides in a significant number of RA patients. Interestingly, this peptide sequence (in a non-citrullinated form) is included in de Jong’s predicted and tested PG T-cell epitopes in RA patients [42] and was also found to be immunogenic in HLA-DR4 transgenic mice [43]. Regarding the humoral responses, an earlier report [14] identified Abs in RA SF that reacted with human PG. The same study suggested that these Abs in RA SF were specific to the G1 domain of PG [14], but it was not known whether such Abs recognized native or citrullinated epitope(s) within the G1 domain.

Using the in vitro citrullinated rhG1 domain of PG and hCII, we were able to detect Abs specific to CitPG and CitCII in the majority of ACPA+ sera from RA patients. We found moderate but significant correlations of the serum levels of anti-CitPG Abs with the levels of Abs to CCP. The correlation between anti-CitPG and anti-CitCII was higher, suggesting that in vivo citrullination of cartilage matrix molecules may contribute to ACPA production in RA patients. However, both anti-CitPG- and anti-CitCII-specific Abs were present at much lower levels in the patients’ sera than those against MCV or CCP3, indicating that autoAbs directed to CitPG and/or CitCII represent a relatively small proportion of the ACPA pool, and may arise as a result of epitope spreading to citrullinated self-proteins during the course of RA [8][44][45].

To our knowledge, our study is the first to show the presence of ACPA-reactive citrullinated human PG epitopes and to demonstrate humoral immunity against CitPG in RA patients. CitPG (and other citrullinated matrix molecules) in cartilage are likely accessible by ACPA present in the serum and/or SF of RA patients with joint inflammation. Because ACPA is not typically produced by healthy individuals or patients with OA, the presence of CitPG in normal or OA articular cartilage may remain unnoticed by the immune system. As reported by Law *et al* [12], some healthy individuals carrying the “shared epitope” in the HLA-DR*0401 allele exhibit T-cell reaction to an epitope in CitPG, but it is much lower in magnitude than the T-cell response of RA patients to the same epitope. It is likely that, even if T-cell reactivity to CitPG is present in healthy or OA subjects, it does not reach the threshold required for B-cell help and autoAb production, and does not result in pathologic reactions against cartilage in the joints. In contrast, CitPG can become a target of ACPA in RA joints, as CitPG can provoke robust T-cell responses and autoAb production in the case of an insufficiently controlled adaptive immune system. Although the involvement of ACPA in RA joint pathology is unclear, the arthritogenic potential of ACPA and some citrullinated proteins has been demonstrated in animal models of RA [46][47]. Therefore, it is conceivable that immune complexes formed between CitPG and anti-CitPG Abs are able to trigger local inflammatory reactions, thus contributing to the initiation or perpetuation of joint inflammation in ACPA+ RA patients.

## Supporting Information

S1 Fig Amino acid sequence with the arginine residues and immunodominant epitopes depicted in the recombinant G1 domain of human PG aggrecan. Amino acid sequence of the recombinant hG1 protein containing 329 amino acids of the G1 domain and 44 amino acids of the neighboring interglobular domain of human PG aggrecan. Numbering of amino acids starts after the signal peptide. Most or all of the 24 arginine (R, red font) residues (23 in the G1 domain and 1 in the interglobular region) may be converted to citrulline by peptidyl arginine deiminase (PAD) enzymes. The sequences of two confirmed T-cell epitopes [11][12][13] within the G1 domain are highlighted in boldface. The 3-dimensional structure of the G1 domain is shown in Fig 5A. Blue fonts depict the neoepitopes generated by stromelysin (VDIPEN) and aggrecanase (NITEGE) cleavage. The complete amino acid sequence of human PG aggrecan (including the signal peptide and the whole core protein) is found at http://www.uniprot.org/uniprot/P16112#sequences.
(TIF)

## Abbreviations

Ab: antibody
ACPA: anti-citrullinated protein Ab
CCP: citrullinated cyclic peptide
CII: type II collagen
CitCII: citrullinated CII
CitPG: citrullinated PG
hG1: G1 domain of human proteoglycan (aggrecan)
mAb: monoclonal Ab
MCV: mutated citrullinated vimentin
PBS: phosphate-buffered saline
PG: proteoglycan (aggrecan)
OA: osteoarthritis
RA: rheumatoid arthritis
rhG1: recombinant G1 domain of human cartilage PG aggrecan
RF: rheumatoid factor
SF: synovial fluid
