# Supplementary figures and images for: Characterization and Localization of Citrullinated Proteoglycan Aggrecan in Human Articular Cartilage
Source: PLoS One. 2016 Mar 4;11(3):e0150784. doi: 10.1371/journal.pone.0150784 (PMC4778950; doi:10.1371/journal.pone.0150784)

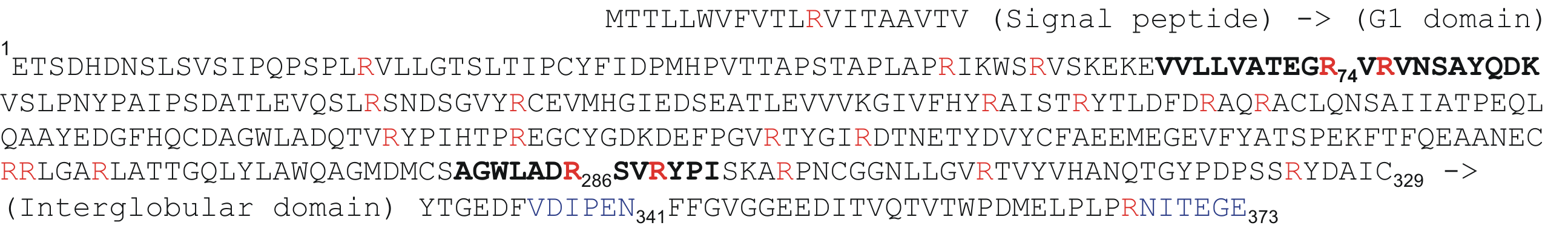

Supplement: S1 Fig — Amino acid sequence of the recombinant hG1 protein containing 329 amino acids of the G1 domain and 44 amino acids of the neighboring interglobular domain of human PG aggrecan. Numbering of amino acids starts after the signal peptide. Most or all of the 24 arginine (R, red font) residues (23 in the G1 domain and 1 in the interglobular region) may be converted to citrulline by peptidyl arginine deiminase (PAD) enzymes. The sequences of two confirmed T-cell epitopes [11][12][13] within the G1 domain are highlighted in boldface. The 3-dimensional structure of the G1 domain is shown in Fig 5A. Blue fonts depict the neoepitopes generated by stromelysin (VDIPEN) and aggrecanase (NITEGE) cleavage. The complete amino acid sequence of human PG aggrecan (including the signal peptide and the whole core protein) is found at http://www.uniprot.org/uniprot/P16112#sequences. (TIF) [file pone.0150784.s001.tif]
